# The tight junction protein occludin modulates blood–brain barrier integrity and neurological function after ischemic stroke in mice

**DOI:** 10.1038/s41598-023-29894-1

**Published:** 2023-02-18

**Authors:** Shintaro Sugiyama, Tsutomu Sasaki, Hiroo Tanaka, Haomin Yan, Takeshi Ikegami, Hideaki Kanki, Kumiko Nishiyama, Goichi Beck, Yasufumi Gon, Shuhei Okazaki, Kenichi Todo, Atsushi Tamura, Sachiko Tsukita, Hideki Mochizuki

**Affiliations:** 1 Department of Neurology, Graduate School of Medicine, Osaka University, Yamadaoka 2-2, Suita, Osaka 565-0871 Japan; 2 Advanced Comprehensive Research Organization, Teikyo University, Itabashiku, Tokyo 173-0003 Japan; 3 Laboratory of Barriology and Cell Biology, Graduate School of Frontier Biosciences, Osaka University, Suita, Osaka 565-0871 Japan; 4 Department of Pharmacology, Teikyo University School of Medicine, Itabashi-Ku, Tokyo, 173-8605 Japan

**Keywords:** Neuroscience, Blood-brain barrier

## Abstract

Blood–brain barrier (BBB) disruption contributes to brain injury and neurological impairment. Tight junctions (TJs) and cell–cell adhesion complexes develop between endothelial cells in the brain to establish and maintain the BBB. Occludin, the first transmembrane protein identified in TJs, has received intense research interest because numerous in vitro studies have suggested its importance in maintaining BBB integrity. However, its role in maintaining BBB integrity after ischemic stroke is less clear owing to the lack of in vivo evidence. This study aimed to investigate the dynamics and function of occludin across the acute and chronic phases after stroke using occludin-deficient mice. By photochemically induced thrombosis model, the expression of occludin was decreased in brain endothelial cells from ischemic lesions. The neurological function of occludin-deficient mice was continuously impaired compared to that of wild-type mice. BBB integrity evaluated by Evans blue and 0.5-kDa fluorescein in the acute phase and by 10-kDa fluorescein isothiocyanate-labeled dextran in the chronic phase was decreased to a greater extent after stroke in occludin-deficient mice. Furthermore, occludin-deficient mice showed decreased claudin-5 and neovascularization after stroke. Our study reveals that occludin plays an important role from the acute to the chronic phase after ischemic stroke in vivo.

## Introduction

Stroke remains the fifth leading cause of death, with 795,000 cases annually, 87% of which are cerebral infarctions^1^. In addition, 2.4% of the U.S. population is disabled by stroke^2^. Dysfunction of the blood–brain barrier (BBB) is associated with the pathogenesis of many neurological diseases, including stroke^3^. In cerebral infarction, ischemia and reperfusion disrupt the BBB, leading to reperfusion injury and hemorrhagic changes^4^.

Tight junctions (TJs), a cell–cell adhesion complex, develop robustly between brain endothelial cells to form a paracellular barrier^5^, limiting the free diffusion of substances between the blood and brain and establishing and maintaining a BBB^6^. Endothelial TJs are a supramolecular complex composed of transmembrane proteins, such as the claudin family members, occludin, tricellulin, and junctional adhesion molecules; as well as scaffold proteins, such as zonula occludens (ZO)-1 and ZO-2^7–10^. Among these, claudin-5 is considered a key structural and functional component of the BBB^11–14^.

In addition to claudin-5, occludin is of significant research interest because it is a widely used marker of BBB integrity^15–17^. Accumulating in vitro studies using cultured endothelial and epithelial cells have suggested the importance of occludin in BBB integrity^18–23^. However, the role of occludin in BBB integrity has not yet been determined in occludin-deficient mice, and further in vivo experiments are required.

Using occludin-deficient mice, the present study elucidated how occludin modulates BBB integrity and neurological function after ischemic stroke. Furthermore, the results suggest that decreased expression of occludin might be associated with the exacerbation of cerebral infarction. Our findings confirm how the expression level of occludin may be indicative of BBB integrity, which may lead to new treatments for neurological disorders that target occludin.

## Results

### Expression of occludin, claudin-5, and ZO-1 decreased after photothrombotic stroke

**Figure 1:**
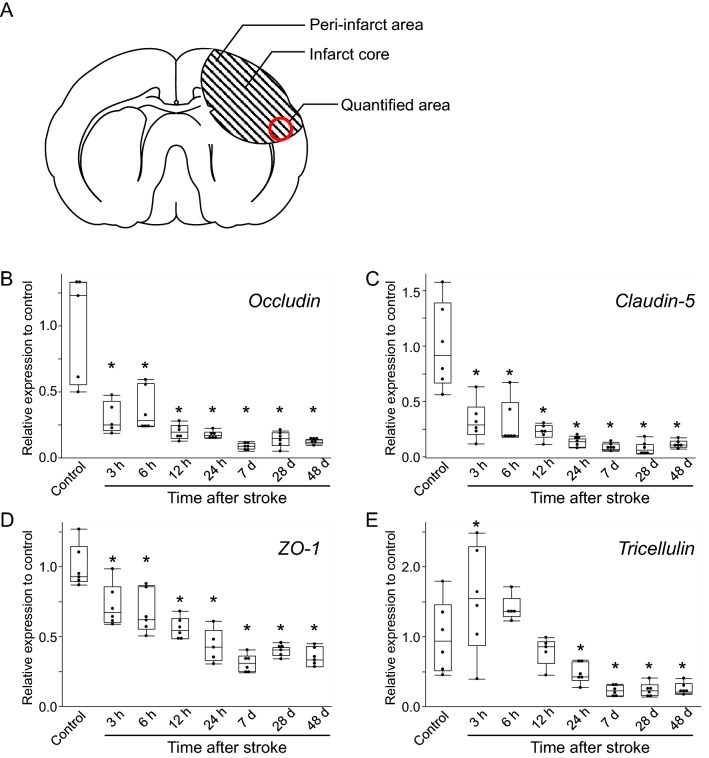
mRNA expression level of tight junction proteins of the blood–brain barrier after cerebral infarction. Quantification of mRNA expression was performed using samples collected from the peri-infarct area shown in (**A**). Box and whiskers with the maximum and minimum relative expression levels of occludin (**B)**, claudin-5 (**C**), ZO-1 (**D**), and tricellulin (**E**) are shown. The expression levels of occludin, claudin-5, and ZO-1 are decreased after 3 h. The expression of tricellulin is decreased after 24 h. The mRNA expression is normalized to that of β-actin (ΔCt = Ct target–Ct actin). Dunnett’s test is performed. *P < 0.05 vs control. n = 5–6 each.

Several studies using rat models have shown that the expression of occludin in brain endothelial cells decreases after stroke^19,24–26^. However, little is known about the spatiotemporal expression of occludin during ischemic stroke in a mouse model. In our study, quantitative real-time PCR analyses revealed that, in wild-type mice, the mRNA level of occludin, as well as those of claudin-5 and ZO-1, decreased starting from the acute phase at 3 h after stroke, and the decline continued into the chronic phase 48 days after stroke (Fig. 1B–D). Occludin and claudin-5 mRNA decreased rapidly 3 h after stroke onset (Fig. 1B,C), while ZO-1 mRNA decreased gradually (Fig. 1D). Furthermore, the mRNA level of tricellulin, a major component of tricellular TJs in brain endothelial cells^21,27–29^, was maintained until 6 h after stroke, after which it gradually decreased (Fig. 1E). The quantified areas of mRNA level are illustrated in Fig. 1A.

**Figure 2:**
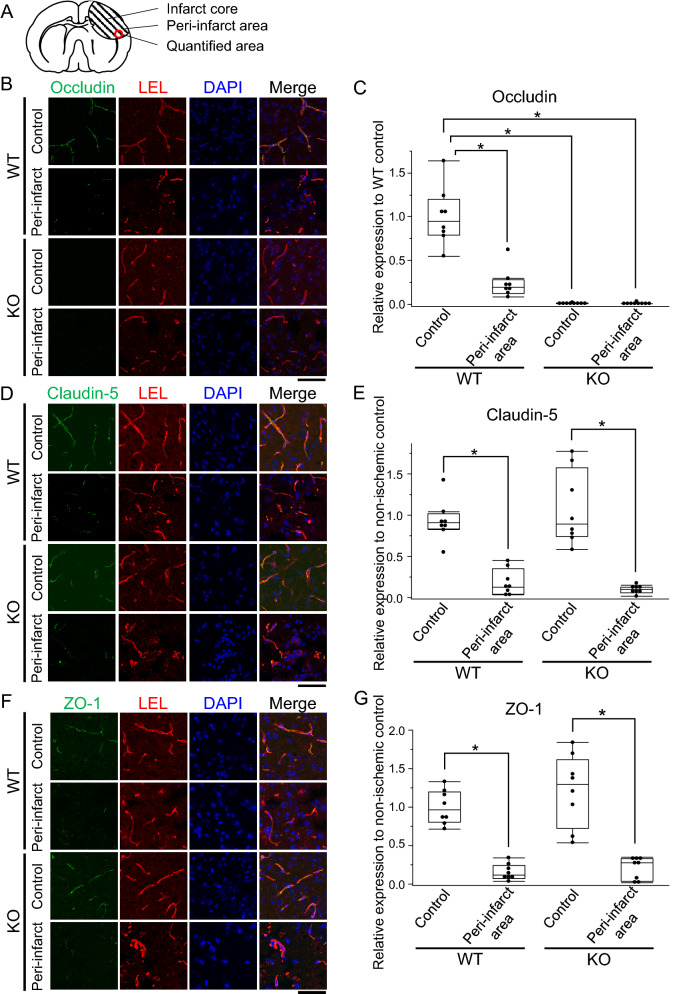
Immunofluorescence analysis of occludin, claudin-5, and ZO-1 24 h after cerebral infarction in wild-type mice. Images and quantification for immunofluorescence analysis were obtained from samples collected from the peri-infarct area shown in (**A**). Occludin (**B**), claudin-5 (**D**), and ZO-1 (**F**) expressions are decreased in the ischemic lesion 24 h after PIT. (**C**–**G**) show box and whiskers with maximum and minimum of the relative area of each protein to wild-type control. Claudin-5 (**D** and **E**) and ZO-1 (**F** and **G**) also decreased at 24 h after stroke compared to physiological controls. Student’s t-test is performed; *P < 0.05. Scale bar = 100 µm. n = 8 each. LEL: lycopersicon esculentum lectin, PIT; photochemically induced thrombosis.

Next, to examine the spatial expression changes of occludin during ischemia, we performed immunofluorescence analyses for occludin and lycopersicon esculentum lectin (LEL), a marker of brain endothelial cells. The quantified areas of immunofluorescence analyses are shown in Fig. 2A. Occludin was highly expressed in the brain endothelial cells of wild-type mice before stroke induction (Fig. 2B). At 24 h after stroke, the expression level of occludin was decreased in the brain endothelial cells of ischemic lesions (Fig. 2B and C). Furthermore, expression of claudin-5 (Fig. 2D and E) and ZO-1 (Fig. 2F and G) also decreased in the peri-infarct area at 24 h after stroke.

Thus, analysis of the spatiotemporal dynamics of occludin expression showed that it decreased, as did claudin-5 and ZO-1, in brain endothelial cells from ischemic lesions after stroke in wild-type mice. The post-ischemic dynamics of occludin suggest its importance to the integrity of the BBB and neurological function after ischemic stroke.

### Occludin-deficient mice exhibited increased infarction volume, BBB dysfunction, and deteriorated neurological function after stroke

**Figure 3:**
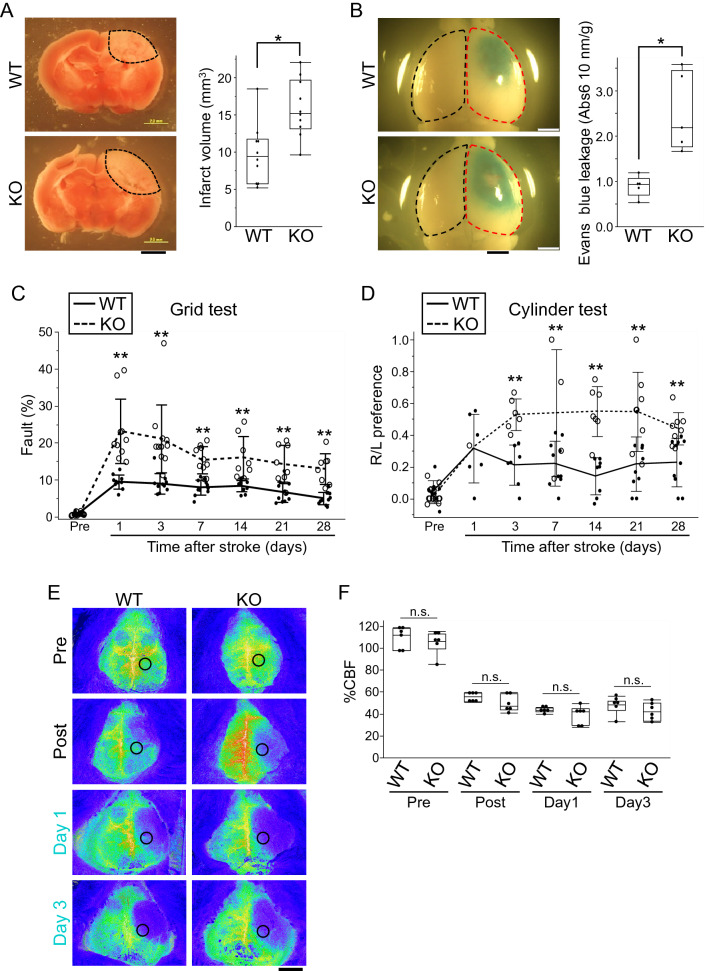
Increased infarction volume and leakage and deteriorated neurological function in occludin-deficient mice. In the acute phase, the infarct volume (**A**) is larger in wild-type mice(n = 10) than in occludin-deficient mice(n = 10). The infarct volume of the area indicated by the black dotted line in A was quantified. Leakage of Evans blue (**B**) is higher in occludin-deficient mice (n = 5) than in wild-type mice (n = 5). Evans blue leakage was quantified in the infarcted hemisphere shown by the red dotted line and in the non-infarcted hemisphere shown by the black dotted line (**B**). The motor function of occludin-deficient mice (n = 12) was worse than that of wild-type mice (n = 13) in the grid test (**C**) and the cylinder test (**D**) from the acute to the chronic phase. The cerebral blood flow (CBF) images are shown in (**E**), and the box and whiskers with max and min of CBF is shown in (**F**). CBF was similar at each time point between wild-type mice and occludin-deficient mice. CBF was quantified in the area indicated by the black circle (**E**). An unpaired t-test was performed. *P < 0.05. Scale bar = 2 mm.

To directly investigate the role of occludin in the pathophysiology of stroke, the infarct volume, BBB integrity, and neurological function post-stroke were assessed in occludin-deficient mice. The infarct volume in occludin-deficient mice 48 h after stroke was significantly larger than that in wild-type mice (Fig. 3A). Furthermore, to determine the BBB integrity of occludin-deficient mice after stroke, extravascular leakage of Evans blue dye was evaluated 24 h after stroke, showing greater leakage in occludin-deficient than in wild-type mice (Fig. 3B).

According to the results of the grid test and cylinder test, neurological function in occludin-deficient mice was significantly worse than that of wild-type mice from the early phase to the chronic phase after stroke (Fig. 3C and D). Moreover, occludin-deficient mice had a higher post-stroke mortality rate than did wild-type mice (Table 1). Measurement of CBF immediately before and after stroke, and at 1 and 3 days after stroke, showed that the decrease in CBF throughout the infarcted hemisphere (excluding infarct lesions) was similar between the wild-type mice and the occludin-deficient mice (Fig. 3E and F).

**Table 1:** Mortality rate after cerebral infarction.

	Wild-type mice	Occludin-deficient mice
Number of mice subjected to PIT	12	13
Number of alive mice at 28 day	11	8
Mortality rate (%)	8.3	38.5

*PIT*: Photochemically Induced Thrombosis stroke model.

Collectively, these findings support the idea that occludin deficiency causes increased infarct volume and BBB dysfunction, as well as deteriorated neurological function after photothrombotic stroke in mice.

### Occludin-deficient mice exhibited lower claudin-5 and ZO-1 expression in brain endothelial cells after stroke

**Figure 4:**
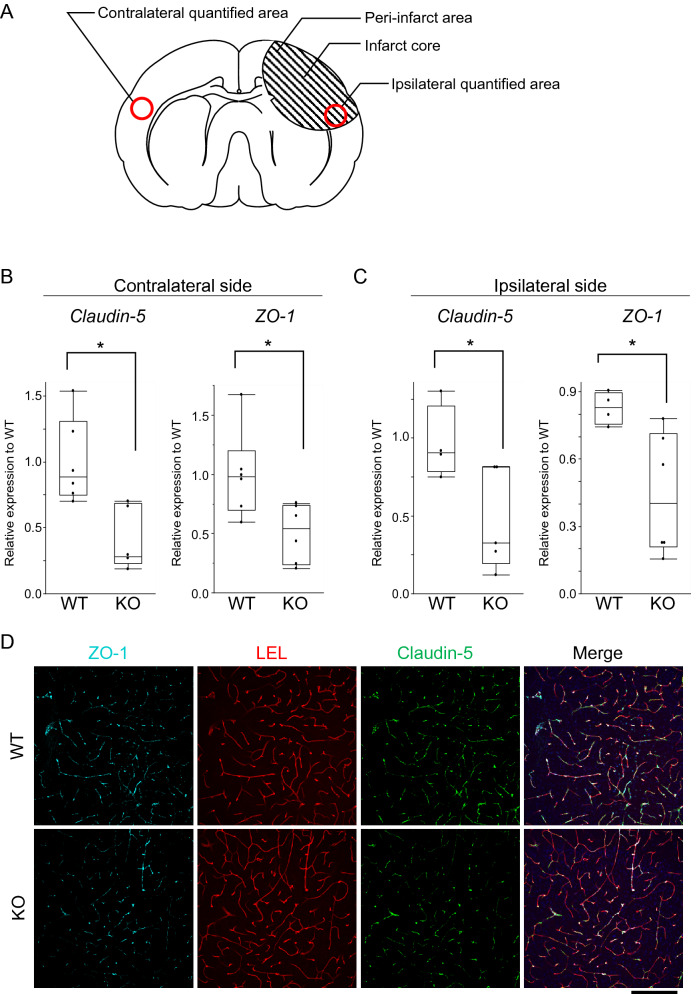
Sections of 30-µm thickness and mRNA expression level of tight junction proteins. The mRNA expression levels were quantified in the peri-infarct area shown in (**A**). The expression levels of ZO-1 and claudin 5 are decreased in occludin-deficient mice (**B**–**D**). The box and whiskers with maximum and minimum of relative mRNA expression levels in wild-type mice and occludin-deficient mice are shown in (**B** and **C**). The mRNA expression levels of claudin-5 and ZO-1 in the contralateral side of the cerebral infarction (left: claudin-5; right: ZO-1) and on the ipsilateral side (left: claudin-5; right: ZO-1) are decreased. Immunofluorescent analyses of 30-µm thick sections are shown in (**D**). Unpaired t-tests are used. *P < 0.05. Scale bar = 200 µm. n = 4–6 each. LEL: lycopersicon esculentum lectin.

Given that occludin-deficient mice with experimental stroke exhibited more severe BBB dysfunction, which might lead to increased infarct volume and deteriorated neurological function, we investigated the expression levels of claudin-5 and ZO-1, which are key structural and functional components of the BBB. Quantitative real-time PCR analyses revealed that claudin-5 and ZO-1 mRNA levels were lower both contralaterally and ipsilaterally to the infarction in occludin-deficient mice than in wild-type mice (Fig. 4A–C). Consistent with these results, immunofluorescent analyses of thick brain sections showed that claudin-5 and ZO-1 signals in brain endothelial cells tended to be lower in occludin-deficient mice after stroke than in wild-type mice (Fig. 4D).

It is possible that the more severe BBB dysfunction in occludin-deficient mice after stroke is due to the lower expression levels of claudin-5 and ZO-1 in the brain endothelial cells.

### Occludin-deficient mice exhibited more severe BBB dysfunction in the chronic phase after stroke

**Figure 5:**
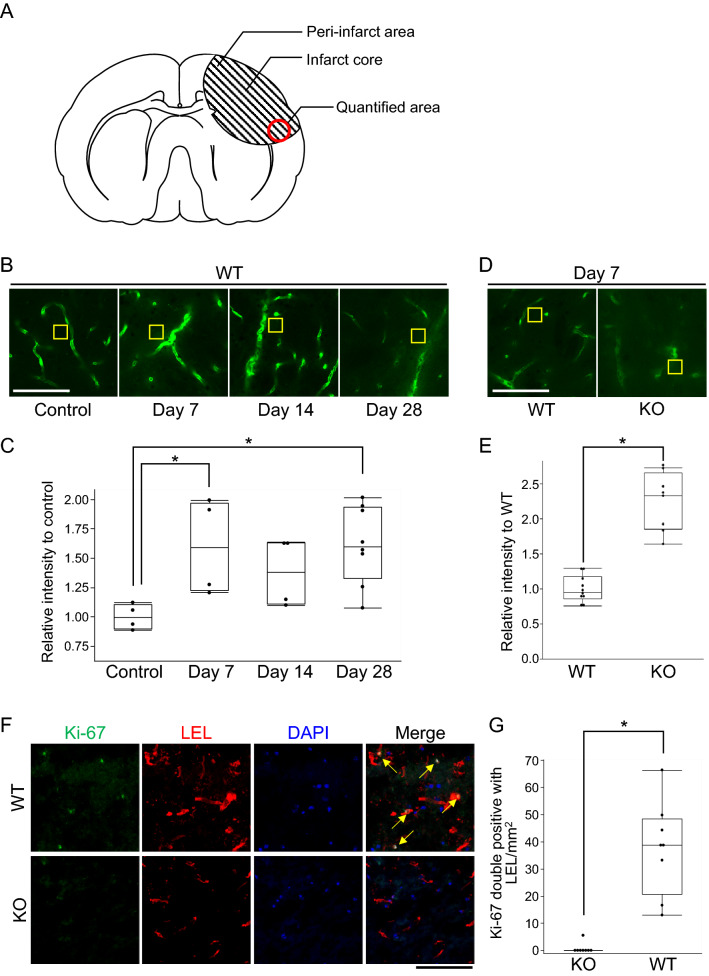
Extravascular leakage of FD-10 and expression of Ki-67 in chronic stage of cerebral infarction. FD-10 leakage and the number of Ki-67 cells merged with lycopersicon esculentum lectin (LEL) were quantified in the peri-infarct area shown in (**A**). FD-10 leakage is also observed in the chronic phase after infarction in wild-type mice (n = 4 each) (**B**). The relative intensity of FD-10 to the control is shown in the box and whiskers with maximum and minimum (**C**). This intensity is significantly increased on days 7 and 28. Fluorescence measurements relative to controls. One-way ANOVA followed by Dunnett’s post hoc test for multiple comparisons (**C**). The extravascular leakage of FD-10 on 7 day (**D**) is significantly higher in occludin-deficient mice (n = 4) than in wild-type mice (n = 5). The relative intensity in comparison to that in wild-type mice is shown in the box and whiskers with maximum and minimum (**E**). The yellow squares in (**B** and **D**) indicate representative measurement areas. The number of Ki-67 cells merged with LEL per area in the infarcted area was higher in wild mice (n = 4) than in occludin-deficient mice (n = 4) on day 7 after cerebral infarction (**F**). The arrows indicate that Ki-67 merged into the LEL. Box and whiskers with maximum and minimum are shown (**G**). Dunnett’s test is performed in (**C**). Unpaired t-test are performed in (**E** and **G**). Scale bar: 100 µm. *P < 0.05. FD-10: fluorescein isothiocyanate-dextran with average molecular weight of 10, 000 Da.

Decreased neurological function in occludin-deficient mice after ischemic stroke continued until the chronic phase (Fig. 3C and D). In wild-type mice, the decreased BBB integrity against FD-10 (molecular weight = 10 kDa) was prolonged from the subacute phase (7 days) to the chronic phase (14 and 28 days) after ischemia (Fig. 5A–C). When FD-10 leakage was compared between wild-type and occludin-deficient mice in the subacute phase (7 days), extravasation of FD-10 was still significantly more enhanced in occludin-deficient mice (Fig. 5A,D and E).

Given that occludin-deficient mice exhibited more severe BBB dysfunction under ischemic conditions than wild-type mice (Figs. 3B and 5D), we considered the possibility that they may exhibit BBB deficiency under physiological conditions. However, the extravasation of fluorescein (molecular weight = 0.5 kDa) (Fig. S1B and C) and FD-4 (molecular weight≒4 kDa) (Fig. S1D and E) did not differ between occludin-deficient mice and wild-type mice at 3 min, 30 min, 1 h, and 3 h after administration. In addition, the extravasation of biotin (molecular weight = 0.5 kDa) was similar between occludin-deficient mice and wild-type mice under physiological conditions (Fig. S1F and G). The quantified areas of leakage are shown in the schema of Fig. S1A.

Given that occludin is reportedly involved in cell proliferation and apoptosis, we examined its effect on angiogenesis^30–32^ by analyzing Ki-67 expression in brain endothelial cells after stroke. The number of Ki-67 and LEL double-positive cells was significantly lower after stroke in occludin-deficient mice than in wild-type mice. (Fig. 5A,F and G). These findings suggest that occludin is associated with angiogenesis following cerebral infarction.

## Discussion

Occludin is a TJ protein and a key structural and functional component of the BBB. Similarly to claudin-5, its decrease in brain endothelial cells is an indicator of BBB disruption^15–17^. The important contribution of occludin in maintaining BBB integrity is based on numerous in vitro studies, but its precise role in occludin-deficient mice remains unclear^18–23^. Our occludin-deficient mouse study shows that a decrease in occludin exacerbates neurological deterioration, accompanied by an expansion of infarct volume and BBB dysfunction after cerebral infarction. The decreased expression of occludin continuously induced BBB dysfunction from the acute to the chronic phase after ischemic stroke.

In the present study, it was unclear whether occludin deficiency directly induced BBB dysfunction after stroke. We considered two possibilities: one is that occludin deficiency directly decreases claudin-5 and ZO-1, and the other is that occludin deficiency exacerbates tissue damage, including BBB destruction, after stroke, which secondarily leads to more severely reduced claudin-5 and ZO-1. In support of the former is a paper that states that for occludin to localize to the TJ, it binds to the underlying cytoskeleton via ZO-1^33^, and that the presence of occludin affects ZO-1 expression in vitro^34^. There are also studies showing that claudin-5 forms cis-oligomerization with TAMPs under physiological conditions^35^. On the other hand, the occludin deficiency could have indirectly induced BBB dysfunction by decreasing claudin-5 and ZO-1 levels as a result of major tissue damage. Cerebral ischemia similarly decreases the expression levels of TJ proteins such as occludin, ZO-1, Claudin-5, and JAM-A. On the other hand, many reports indicate that suppression of MMPs and cytokines similarly restores these TJ protein levels^26,36–38^. Few studies have examined the effects of directly regulating occludin expression on cerebral infarction. The occludin-deficient mice exhibited more severe BBB dysfunction under ischemic conditions in this study, however, it remains unclear what molecules are leaked via TJs in occludin-deficient mice. Matrix metalloproteinases (MMPs) also disrupt the blood–brain barrier (BBB) during ischemia-reperfusion^26,36^. There is also accumulating evidence for an involvement of cytokines^37^ or chemokines^38^ in postischemic BBB disruption. Molecules with increased leakage through barriers due to the loss of occludin in TJs may promote the release of these MMPs, inflammatory cytokines, and other molecules. More recently, the study in the intestinal epithelium of occludin KO mice have shown that occludin affects proinflammatory cytokines^32^. Further investigation is needed to determine what mechanisms by which occludin deficiency reduces claudin-5 and ZO-1, and what molecules are enhanced leakage through the TJ barrier due to occludin deficiency.

Interactions between tight junction-associated marvel proteins (TAMPs)^39^, consisting of occludin, tricellulin, and marvelD3, and claudins have been shown in living TJ-free HEK 293 cells under physiological conditions^35^. A strong interaction or oligomerization of TAMPs with several claudins has been demonstrated, which means that claudins form the central backbone of the TJ strands. Meanwhile, TAMPs support the TJ strand network, allowing them to take on their typical physiological morphology^35^. The interactions with claudins also determine the dynamic properties of TAMPs, such as their membrane mobility, binding properties, and localization^35^. Therefore, the molecular mechanism underlying BBB deficiency after stroke in occludin-deficient mice remains unclear, and further studies are needed.

Occludin-deficient mice show complex phenotypes, such as gastritis, male infertility, calcification in the brain, thinning of the compact bones, deafness, and lactation failure^40–43^. However, the paracellular barrier functions of gastric and mammary epithelial TJs remain intact^42,43^. Several in vitro studies have shown that occludin knockdown or knockout does not change the steady-state paracellular barrier functions of TJs^39,44,45^. In contrast, a recent report revealed that occludin knockout in cultured epithelial cells impaired the paracellular barrier function of TJs, albeit in a limited capacity^21^. This inconsistency might be due to the differences between in vivo and in vitro studies, as well as in the cell types assayed. Thus, further studies are needed to comprehensively understand the contribution of occludin to the paracellular barrier function of TJs.

Unlike occludin, claudin-5, and ZO-1, which rapidly decreased early after ischemia, tricellulin remained expressed until 6 h after ischemia before gradually declining. Tricellulin is highly distributed across the tricellular TJs of epithelial and endothelial cells^21,27–29^. However, it is also present in bicellular TJs when occludin is knocked down or knocked out^40,46^. This may compensate for the decrease in occludin expression after infarction. Specifically, the interactions and importance of occludin and tricellulin may differ between epithelial and endothelial cells in vivo; therefore, further studies are needed. As occludin and tricellulin are jointly involved in the formation of TJs and maintaining barrier function^21^, we believe that they may both be downregulated in the long term, which could have caused the impaired barrier function and exacerbation of neurological dysfunction observed in our present study.

A novel role for occludin in neovascularization and angiogenesis has recently been reported^31^. Occludin has also been shown to play a crucial role in proliferation, sprouting, and tube formation of late endothelial progenitor cells^31^. Consistent with these reports, we found that occludin deficiency is associated with decreased proliferation of brain endothelial cells after stroke, which might lead to the long-term exacerbation of neurological dysfunction following stroke.

In conclusion, occludin deficiency increases BBB permeability, exacerbates tissue damage, and decreases angiogenesis after cerebral infarction, leading to the long-term worsening of functional recovery following stroke in mice. These findings suggest that targeting occludin, in addition to claudin-5, may be a novel treatment strategy for neurological disorders. In addition, our findings clearly show that the expression of occludin is a more reliable indicator of BBB integrity.

## Methods

### Antibodies

For immunohistochemical analyses, antibodies against claudin-5 antibody (Alexa Fluor 488, Invitrogen, Carlsbad, CA) were used at a 1:500 dilution; claudin-5 (Abcam; Cambridge, UK) was used at a 1:1000 dilution; ZO-1 (Invitrogen) was used at 1:500 or 1:1000 dilution; LEL and DyLight 594 (Vector Laboratories; Burlingame, CA) were used at 1:500 or 1:4000 dilution; and 4′,6-diamidino-2-phenylindole was used at a 1:1000 dilution. Antibodies targeting occludin (MOC37) were raised as previously described^47^.

### Mice

Occludin-deficient mice were generated as described by Saitou et al.^48^ Adult (9–12 weeks) wild-type mice (C57BL/6 J) were purchased from Jackson Laboratory, Japan (Yokohama, Japan). Occludin-deficient mice were backcrossed to C57Black/6 mice more than 10 times. In total, 218 experimental mice (115 males and 103 females) were used in this study. All experiments were conducted in a double-blinded manner, with different experimenters and experiment evaluators. We randomly allocated animals to various groups using a coin flip approach. The investigators who established the models, performed the behavior tests, and analyzed the data were blinded to group allocation.

### Photochemically induced thrombosis model

Photochemically induced thrombosis** (**PIT) leading to cerebral infarction was developed according to the method we had previously reported^49^. Adult male and female mice aged 8–12 weeks were anesthetized with isoflurane in an induction chamber with 100% oxygen. The skin along the midline of the scalp, from the eye level down to the neck, was incised, and the skull was exposed. Photothrombotic vascular occlusion was induced by intraperitoneal injection of 0.2 mL rose bengal (10 mg/mL) for 5 min. Next, 20-min illumination was applied to the exposed skull using a cold light source (4.5 mm diameter fiber optic end; Hamamatsu Photonics K.K., Hamamatsu, Japan) placed 2.2-mm laterally to the left of the bregma. The illuminated areas included the motor (M1) and somatosensory (S1) cortices. After 20 min of illumination, the scalp skin was closed using surgical cyanoacrylate glue.

### Quantitative real-time polymerase chain reaction

Total RNA was extracted using the ReliaPrep™ RNA Tissue Miniprep System (Promega, Madison, WI). cDNA was prepared by reverse transcription of 100 ng of total RNA using the SuperScript VILO cDNA Synthesis Kit (Invitrogen). The resultant cDNA was used for quantitative real-time polymerase chain reaction (PCR) analyses using Power SYBR Green PCR Master Mix (Invitrogen). One percent of the reverse transcription products and standard plasmids were subjected to quantitative real-time PCR analysis (QuantStudio 7 Flex Real-Time PCR Systems; Thermo Fisher Scientific, Waltham, MA) using mouse β-actin as an internal control. The PCR program was as follows: 10 min of denaturation at 95 °C, followed by 40 cycles of 95 °C for 15 s and 60 °C for 1 min. Quantitative real-time PCR primers for claudin-5, occludin, and ZO-1 are listed in Supplementary Table 1 and were used as previously described^50^.

### Immunohistochemistry

For the fresh frozen section study (Fig. 2B, D, F, and Fig. 5F), brains were frozen on dry ice, sliced at a thickness of 14 µm, pasted on glass slides, and fixed in methanol at −20 °C for 10 min. The slides were incubated in phosphate-buffered saline (PBS) containing 10% normal donkey serum at room temperature for 10 min. The sections were then incubated with the appropriate primary antibody for 30 min at room temperature. Subsequently, the sections were incubated with secondary donkey antibodies conjugated with Alexa Fluor 488 or 594 (1:250; Invitrogen) for 30 min at room temperature. Sections were then mounted with VECTASHIELD (Vector Laboratories) and visualized or photographed using a confocal microscope (LSM-710; Zeiss, Oberkochen, Germany).

In the thick section study (Fig. 4D), the thoracic cavity was exposed under isoflurane anesthesia, and 1% Paraformaldehyde (PFA) was refluxed from the left heart cavity using a peristatic pump for 10 min. The brain was then fixed in 1% PFA at 4 °C after removal, and sliced to 150 µm-thickness with a vibrating blade microtome (VT1000S; Leica Biosystems, Wetzlar, Germany). The slices were blocked with PBS containing 0.2% Triton X and Block Ace (10 mg/mL, DS Pharma Biomedical, Tokyo, Japan) for 5 h at 4 °C. They were then incubated with the primary antibody diluted in Canget Signal Solution A (TOYOBO, Osaka, Japan) with 0.2% Triton X and Block Ace (4 mg/mL) at 4 °C for 16 h. This was followed by incubation with the secondary antibody diluted in Canget Signal Solution A with 0.2% Triton X and Block Ace (4 mg/mL) conjugated to Alexa Fluor 488 or 647 (1:250; Invitrogen) at 4 °C for 12 h. The slices were then sealed with Fluoroscence Mounting Medium (Agilent Technologies, Santa Clara, CA). The cells were observed using a confocal microscope (SpinSR10; Olympus).

### Determination of infarct volume and measurement of cerebral blood flow

2,3,5-Triphenyltetrazolium chloride staining was used to evaluate infarct volume, as previously described^51,52^. The infarct volume was calculated as: the total contralateral hemisphere volume–the nonischemic ipsilateral hemisphere volume. Cerebral blood flow (CBF) was measured as previously described^52^. Surface CBF was recorded using a laser speckle blood flow imaging system (Omegazone OZ-1; OMEGAWAVE, Fuchu, Japan). After induction of general anesthesia, the skull was exposed through a midline scalp incision. The surface of the skull was wiped clean with saline-soaked gauze prior to recording. Color-coded CBF images were obtained in the high-resolution mode. The mean CBF value was measured in identically-sized regions of interest (900 pixels), located 3 mm posterior and 2.5 mm lateral to the bregma, as previously described^52^.

### Behavioral experiments

The cylinder test and grid walking test were performed to evaluate neurological deficits. The cylinder test was performed as previously reported by Monai et al.^53^ For each test, a mouse was placed inside the cylinder for 5 min and lateralization of limb usage preference was evaluated, where R/L preference = ([R + both] − [L + both])/([R + both]) + [L + both]). The grid walking test was performed as previously described by Stokowska et al.^54^ For each test, a mouse was placed on the grid for 5 min, and the foot fault rate of the left forelimb was determined as foot fault/total foot × 100. Mice that took < 30 steps over the 5 min were excluded.

### Measurement of BBB integrity

To evaluate leakage of Evans blue, the mice were injected with 100 µL of 4% Evans blue (Sigma-Aldrich, St. Louis, MO) intravenously through the tail vein 24 h after PIT. After 1 h, the brains were removed and imaged under microscopy (SZ-12; Olympus, Tokyo, Japan). The brain was then separated into ipsilateral and contralateral hemispheres to the PIT stroke model. Next, each hemisphere was supplemented with 500 µL of formamide, transferred to a 55 °C heat block, and incubated for 24 h to extract Evans blue from the tissues. The furamide/Evans blue mixture was centrifuged to pellet any remaining tissue fragments, and absorbance was measured at 610 nm; 500 µL formamide was used as a blank. The Evans blue extravasated per g tissue was determined.

To evaluate the leakage of fluorescein (Sigma-Aldrich), fluorescein isothiocyanate-dextran (FD)-4 (fluorescein isothiocyanate-dextran, Sigma-Aldrich), and FD-10 (Sigma-Aldrich), the mice were injected with 100 µL of 10 mmol/L fluorescein, 100 mg/mL FD-4, and 100 mg/mL FD-10, respectively, through the tail vein 24 h after PIT. After 3 min, the brains were explanted; fixed with 4% paraformaldehyde overnight at 4 °C; and cryoprotected using a 10, 20, and 30% sucrose gradient. Then, 50 µm frozen sections were cut and mounted with Vector-shield (Vector Laboratories). Analysis of tissue cryosections was performed on the same day using constant exposure settings on a Zeiss confocal microscope (LSM-710). Fluorescence in the tissue sections was quantified and compared using ImageJ. The fluorescence values in tissue sections obtained from animals that had been perfused with PBS only without a tracer were also measured as background controls. All perfusion and tissue analyses were performed by investigators blinded to the treatment administered.

The extent of biotin leakage was assessed using terminal perfusion with Sulfo-N-hydroxysulfosuccinimide (NHS)-Biotin (Thermo Fisher Scientific). Hanks’ balanced salt solution, containing Sulfo-NHS-biotin (0.5 mg/mL), was perfused into the left ventricle for 2 min at a rate of 4 mL/min using a peristatic pump. The explanted brains were fixed; cryoprotected; cut into 14-µm-thick frozen sections; incubated with antibodies against LEL, DyLight 594 (1:500), and streptavidin and Alexa Fluor™ 488 conjugate (1:100) overnight at 4 °C; then mounted and visualized with a confocal microscope, as described above.

### Statistical analysis

Most data are presented as boxes and whiskers and mean ± SD. Between-group comparisons were performed using a 2-tailed unpaired Student’s t-test. Multiple group comparisons were performed using one-way analysis of variance (ANOVA), followed by Tukey–Kramer post-hoc analysis or Dunnett’s test. All statistical analyses were performed using JMP Pro statistical software (V15.1.0). Statistical significance was defined as *P* < 0.05.

### Study approval

The animal experimental protocol was approved by the Institutional Animal Care and Use Committee of Osaka University Graduate School of Medicine. All applicable international, national, and/or institutional guidelines for the care and use of animals were followed. Additionally, our study was in compliance with the ARRIVE guidelines.

## Supplementary Information

Supplementary Information.

## Data Availability

The datasets used and analyzed during the current study are available from the corresponding authors on reasonable request.

## Abbreviations

BBB: Blood–brain barrier
CBF: Cerebral blood flow
FD: Fluorescein isothiocyanate-dextran
LEL: Lycopersicon esculentum lectin
PIT: Photochemically induced thrombosis
PCR: Polymerase chain reaction
TAMPs: Tight junction-associated marvel proteins
TJ: Tight junction
ZO: Zonula occludens
