# Supplementary material for: The tight junction protein occludin modulates blood–brain barrier integrity and neurological function after ischemic stroke in mice
Source: Sci Rep. 2023 Feb 18;13:2892. doi: 10.1038/s41598-023-29894-1 (PMC9938878; doi:10.1038/s41598-023-29894-1)
Supplement: Supplementary file 1 — Supplementary Information. [file 41598_2023_29894_MOESM1_ESM.pptx]

## Slide 1
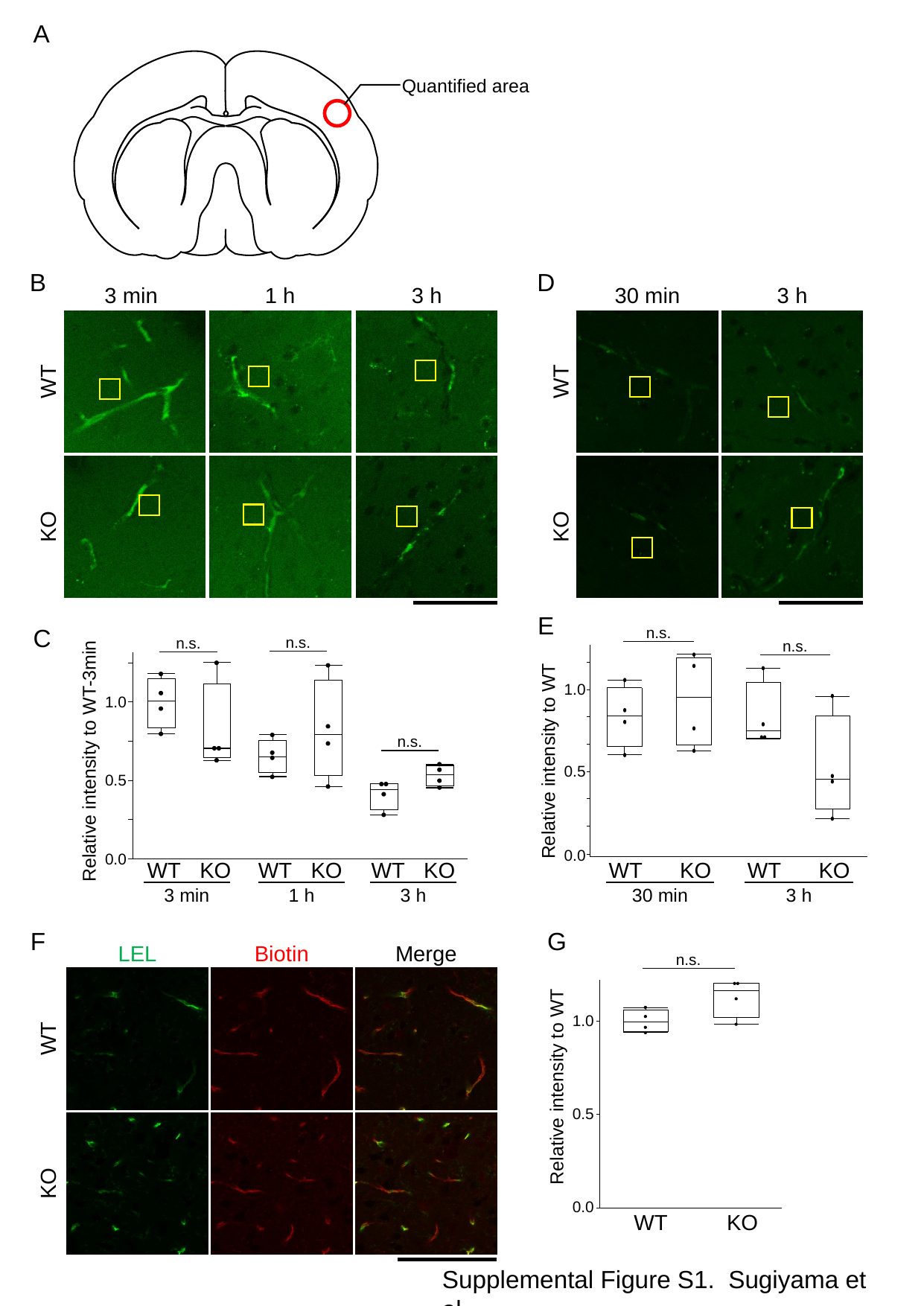

A
Quantified area
B
D
3 min
1 h
3 h
30 min
3 h
WT
WT
KO
KO
E
C
n.s.
n.s.
n.s.
n.s.
1.0
1.0
n.s.
Relative intensity to WT-3min
Relative intensity to WT
0.5
0.5
0.0
0.0
WT
KO
WT
KO
WT
KO
WT
KO
WT
KO
3 min
1 h
3 h
30 min
3 h
F
G
LEL
Biotin
Merge
n.s.
1.0
WT
Relative intensity to WT
0.5
KO
0.0
WT
KO
Supplemental Figure S1. Sugiyama et al.

## Slide 2
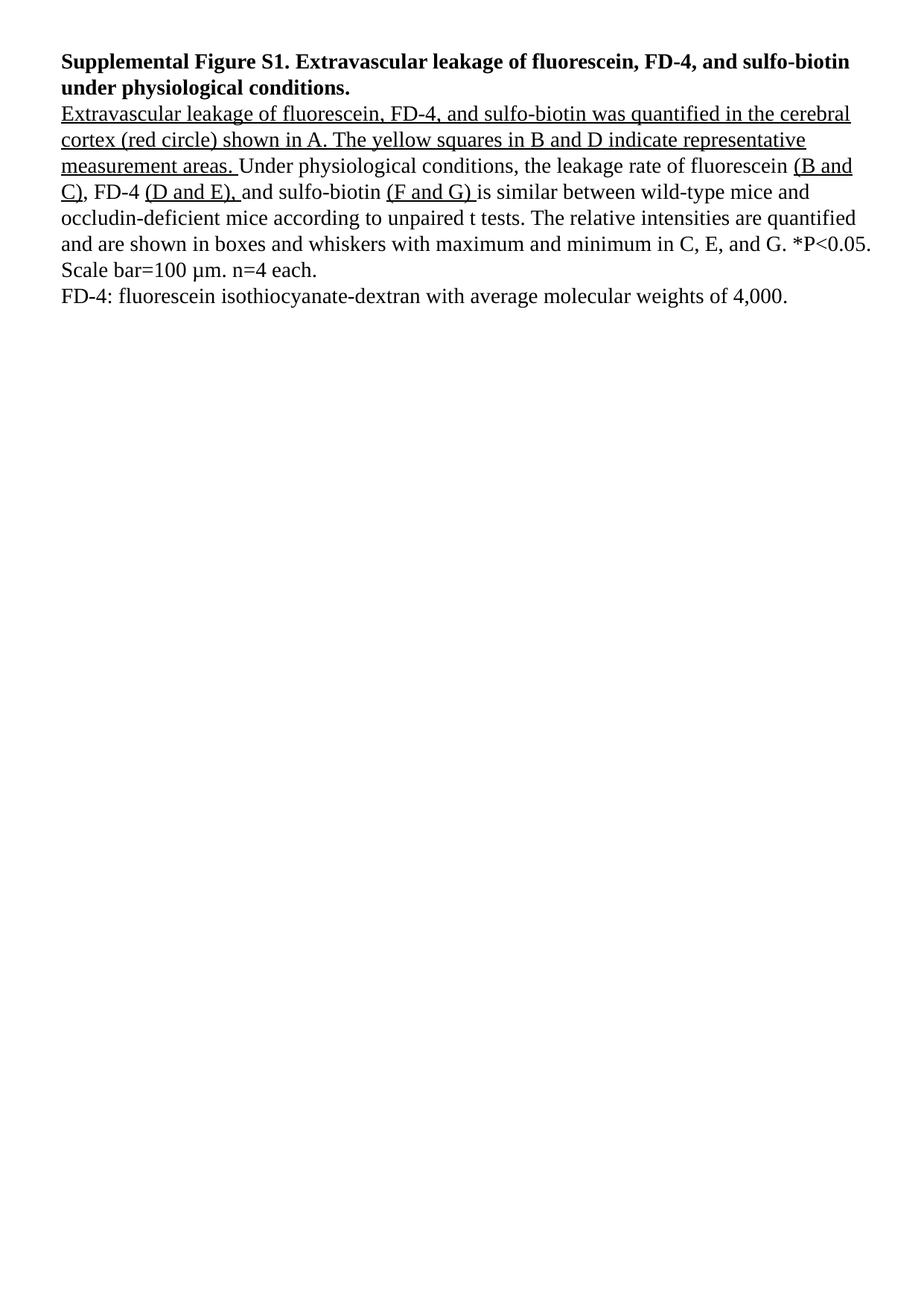

Supplemental Figure S1. Extravascular leakage of fluorescein, FD-4, and sulfo-biotin under physiological conditions.
Extravascular leakage of fluorescein, FD-4, and sulfo-biotin was quantified in the cerebral cortex (red circle) shown in A. The yellow squares in B and D indicate representative measurement areas. Under physiological conditions, the leakage rate of fluorescein (B and C), FD-4 (D and E), and sulfo-biotin (F and G) is similar between wild-type mice and occludin-deficient mice according to unpaired t tests. The relative intensities are quantified and are shown in boxes and whiskers with maximum and minimum in C, E, and G. *P<0.05. Scale bar=100 µm. n=4 each.
FD-4: fluorescein isothiocyanate-dextran with average molecular weights of 4,000.

## Slide 3
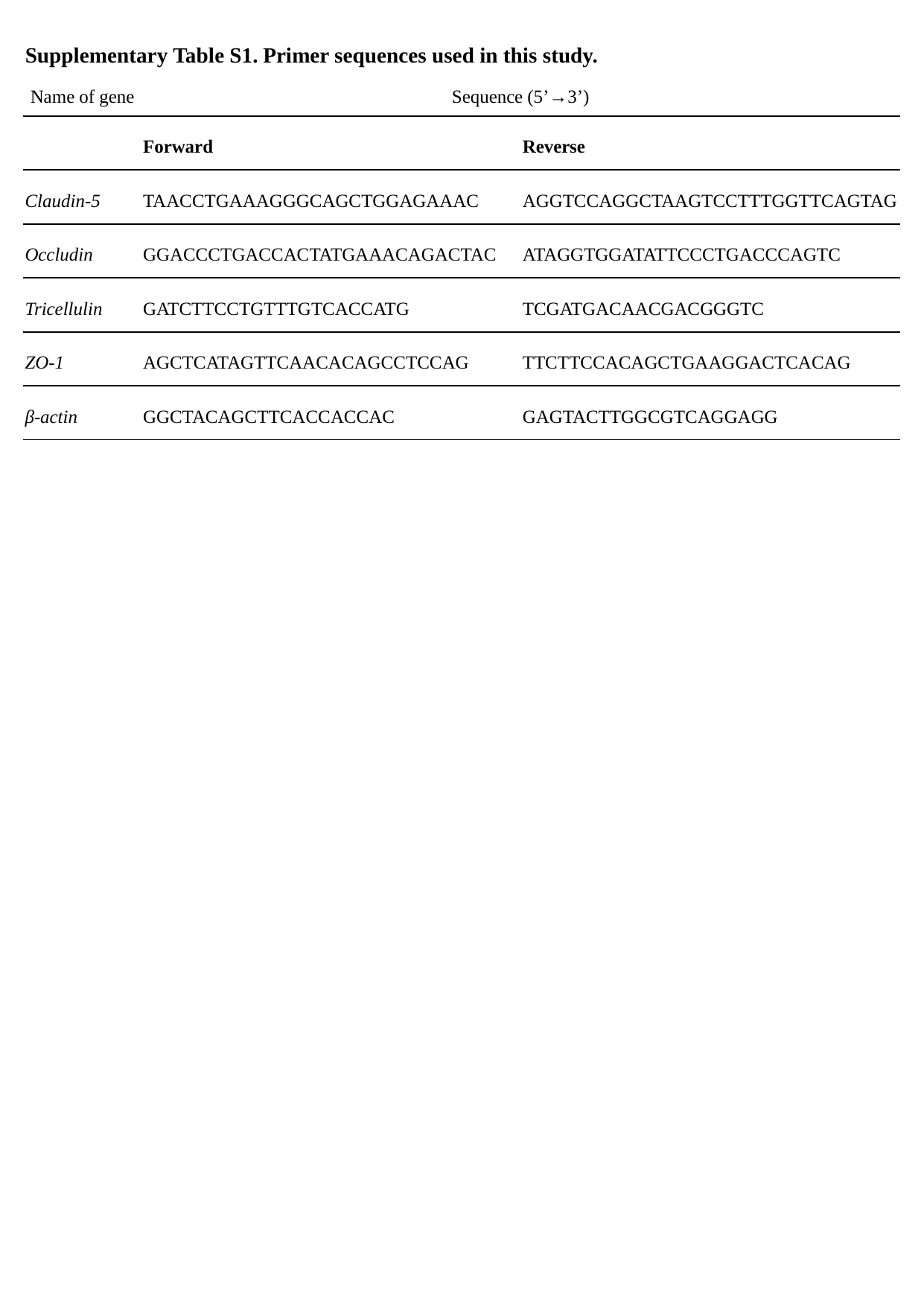

Supplementary Table S1. Primer sequences used in this study.
| Name of gene | Sequence (5’→3’) | |
| --- | --- | --- |
| | Forward | Reverse |
| Claudin-5 | TAACCTGAAAGGGCAGCTGGAGAAAC | AGGTCCAGGCTAAGTCCTTTGGTTCAGTAG |
| Occludin | GGACCCTGACCACTATGAAACAGACTAC | ATAGGTGGATATTCCCTGACCCAGTC |
| Tricellulin | GATCTTCCTGTTTGTCACCATG | TCGATGACAACGACGGGTC |
| ZO-1 | AGCTCATAGTTCAACACAGCCTCCAG | TTCTTCCACAGCTGAAGGACTCACAG |
| β-actin | GGCTACAGCTTCACCACCAC | GAGTACTTGGCGTCAGGAGG |
